# The Book World of Medicine and Science

**Published:** 1903-05-02

**Authors:** 


### The Book World of Medicine and Science.

Aids to Sanitary Science for the Use of Candidates for Public Health Qualifications. By Francis J. Allen, M.D.Edin., D.P.H.Camb., M.O.H. for City of Westminster. Second Edition, revised and edited by R. A. Farrar, M.A., M.D.Oxon., D.P.H Camb. (London: Baillière, Tindall and Cox, 8 Henrietta Street, Covent Garden. 1903. 4s. 6d. cloth; 4s. paper.)

Though primarily intended as a text-book for the use of candidates for Public Health qualifications, and so dealing with much that is technical and statistical, this work contains nevertheless a good deal that the general public might read to their interest and advantage, as, for example, the chapters on Communicable Diseases, Prevention of Infectious Diseases, and Disposal of the Dead. To those contemplating the building or taking of a house, we would recommend the perusal of the chapters on Healthy Sites and Soils (p 113), Drain-testing (p. 54 and p. 111), and House Inspection (p. 125). Also the useful information on such matters as Teale Grates (pp. 79–122), w.c's (p. 47), and Ventilation (p. 98). The book is clearly written, and wherever possible purged of technicalities, and made interesting and comprehensible to the general reader, so will doubtless have a wide circulation beyond those for whom it is intended, especially if the publishers can see their way to issue a cheaper edition shortly.

Cancer: its Causation, and its Curability without Operation. By Robert Bell, M.D.Glasg., F.F.P.S., Consulting Physician to the Glasgow Hospital for Women. Pp. viii. + 271. Crown 8vo. (London: Baillière, Tindall and Cox. 1903. Price 5s. net.)

As the title is almost sufficient to indicate, this work is one which tends to bring English medical literature into disrepute. Dr. Bell in his preface declares that "the longer I have studied the subject of cancer, the more convinced have I become that if it is ever to occupy a place upon the list of curable diseases the public must of necessity be taken into our confidence, be educated up to the point where they will be enabled to recognise not only the disease in its initial stage, but the conditions of life which tend to lead up to its development" The construction and manner of expression leads us to the conclusion that the author intends his book rather for the public than the professional reader. The mystery which still lies around the etiology of cancer, the difficulties connected with its various pathological manifestations, the variations in its clinical indications, the ineffectual character of many of the available methods of treatment, and the dense darkness which makes prognosis but little better than a vague guessing, afford cogent reasons for silent strenuous research into the perplexities of the subject rather than the exploitation of the opinions and methods of an individual. Fortunately, it is not often we are compelled to speak in terms of strong disapprobation of a work written by a medical man, but in the present case, when the first sentence of the book states that "Twenty-six thousand and thirty-five deaths from cancer during the year 1900 is an appalling number, and this is more startling when we consider that 90 per cent. of these deaths might possibly have been avoided," and when almost every chapter, with painful iteration, declares that cancer is remediable if, of course, met with "the remedies which I know exist, and which I have had frequent opportunities of employing with excellent results," we can only express the opinion that the book is not only lacking in all indications of scientific precision, but from its encouragement of a false, unfounded hope is little less than inhuman. It is unnecessary to attempt to deal with the wearisome 41 chapters of this book individually, since almost every one is marked by a want of a true sense of the scientific spirit and stamped by egotism, prejudice, and ignorance. Throughout there is astonishing evidence of the author's lack of accurate pathological knowledge. There is much also in extremely bad taste, as, for instance, where we are informed that "every year hundreds of lives are sacrificed to the callousness and indifference, not to say pigheadedness, of medical men, for which the medical press is largely responsible." Dr. Bell says, "It may seem to my readers presumptuous if I state that the prime object I have in view is to attempt to clear up the pathogeny of malignant disease . . . to invite attention to the intimate pathological and therapeutical relationship which exists between diseased organs and those in sympathy with them." But the whole work is peculiarly devoid of any evidences of what may be termed original work. There are numerous expressions of opinion regarding "the association of uricacidæmia with cancer," the necessity for attacking "the rheumatic element in the blood simultaneously with the treatment of the malignant manifestations," and the advantage of thyroid preparations in cancer; but it may be safely said that such a work as that before us instead of assisting scientific medicine, can prove nothing but a hindrance, and merits the strong disapprobation of all serious students.

A Manual of Toxicology. A Concise Presentation of the Principal Facts Relating to Poisons, with detailed directions for the Treatment of Poisoning. With table of Doses of the Principal and many New Remedies. By Albert H. Brundage, A.M., M.D., Phar.D., Professor of Toxicology, Physiology and Hygiene in the Brooklyn College of Pharmacy, etc. Second edition, revised and enlarged. (Pp. viii. and 375. Crown 8vo. London Baillière, Tindall and Cox. 1902. Price 6s. net.)

The fact that this compact, complete, and convenient little manual has reached a second edition within a year of its first appearance seems to indicate that at least in the land of its birth its merits have not been allowed to pass unrecognised. Certainly in the concise expression of its matter, the practical arrangement in alphabetical order, the clear printing and handy size, the book at once predisposes the reader to regard it favourably. For its capacity it undoubtedly is remarkably full, and without burdening its pages with any redundances, is nevertheless sufficiently complete to meet the necessities of the practitioner, although we are inclined to think that students in this country preparing for certain examinations would not do to rely entirely on such a condensation of essentials. For practical work the volume is likely to prove of real service. For ready reference in emergencies when prompt, intelligent and efficient aid is urgently demanded, it is excellent. It affords a review of the salient points of the subject and to one who has mastered the larger and more exhaustive treatises such a volume as this is not only convenient but actually desirable. Peculiarities in spelling and expression clearly indicate that the book was "made in America," but we are becoming accustomed to the Americanisation of medical as of other matters.

